# Study on Mix Proportion Optimization and Microstructure of Coal-Based Solid Waste (CSW) Backfill Material Based on Multi-Objective Decision-Making Model

**DOI:** 10.3390/ma15238464

**Published:** 2022-11-28

**Authors:** Xinyuan Zhao, Ke Yang, Xiang He, Zhen Wei, Xiang Yu, Jiqiang Zhang

**Affiliations:** 1 State Key Laboratory of Mining Response and Disaster Prevention and Control in Deep Coal Mine, Anhui University of Science and Technology, Huainan 232001, China; 2 Institute of Energy, Hefei Comprehensive National Science Center, Hefei 230031, China

**Keywords:** multi-objective decision model, coal-based solid waste, backfill material, mix proportion optimization, microstructure

## Abstract

The preparation of underground-backfill material from CSW can be used for large-scale disposal of solid waste. The proportion of backfill material plays an important role in transportation and backfilling effect, and the mix-proportion optimization of backfill material is essentially a multi-factor and multi-objective optimization problem. In this paper, to obtain the mix proportion of backfill materials with optimal comprehensive-evaluation indexes, and suitable for the engineering application, the fluidity and strength of backfill material, mainly composed of coal gangue(CG), fly ash (FA), flue gas desulfurization gypsum (FGD gypsum), and gasification coarse slag (GCS), were tested by single-factor transformation method, and the effects of various solid wastes on the slump-flow, bleeding rate and early strength of backfill material were analyzed. The optimal mix proportion of CSW with the slump-flow, bleeding rate, and 3-day and 7-day strengths as the evaluation indicators is FA: GCS: FGD gypsum: CG = 25%:25%:25%:25%, according to the multi-objective decision model. Furthermore, the comprehensive evaluation index that meets the requirements of mine backfilling is obtained by changing the ordinary portland cement (OPC) content, that is, the optimal OPC content is 10% of the total solid waste, and the mass concentration is 78%. Finally, the pore structure, micromorphology, and composition of the backfill material with the optimal mix proportion were studied by Mercury Intrusion Porosimetry (MIP), X-ray Diffraction (XRD), and Scanning Electron Microscope-Energy Dispersive Spectrometer (SEM-EDS). The research results provide a good reference for the field application of CSW for underground backfilling.

## 1. Introduction

In western China, tens of millions of tons of CSWs are discharged from coal power and coal-chemical bases every year, mainly including CG, FA, FGD gypsum, and various waste residues. The stock and increment of CSW are huge, and the utilization rate is low, resulting in a serious shortage of storage capacity in slag yard for solid waste [1,2]. On the other hand, harmful substances, such as heavy metals in CSWs, may leach out, seriously polluting local water, soil, and air, and damaging the ecological environment and people’s health [3,4]. Therefore, the large-scale synergetic utilization of CSW has become an urgent problem faced by the local government.

Backfill mining is a green mining method that backfills general solid waste in solid or fluid form into underground goaf. It is widely used in both coal and non-coal mines in China. This method can not only reduce coal pillars and surface subsidence, but also effectively dispose of solid-waste stockpiles and reduce the adverse effects of solid waste on the ecological environment and public health [5,6,7].

Backfill material is an important part in backfill mining practice, and the mix proportion of backfill material plays a key role in the backfilling effect. Therefore, it is the basis for the application of backfill materials to study reasonable mix proportion and performance [8,9]. Many scholars have studied the mix proportion and performance of mine- solid-waste backfill materials. For example, Lan et al. [10] studied the effect of different admixtures on the properties of backfill materials using experimental methodology; Wu et al. [11] obtained the optimal-backfill-material ratio for the mine based on response-surface methodology and satisfaction-function-coupling theory; Fall et al. [12] proposed and verified the mix-proportion design method of cemented-tailings backfill based on the matching of response-surface methodology and expected-value methodology; Zhang et al. [13] studied the influencing factors and the optimal ratio of solid-waste backfill by orthogonal-test methodology; Li et al. [14] studied the influence of slurry preparation on phosphogypsum-cemented backfill by orthogonal experiment; Wang et al. [15] studied the rheological properties of cemented-paste backfill using uniform-design methodology, and found that mass concentration had the greatest impact on the rheological properties; Wei, Tang et al. [16,17,18] studied the optimal mix proportion and interaction of multi-source CSW using response-surface methodology; Fu et al. [19] studied the influence of mass fraction, OPC content, and aggregate ratio on the unconfined-compressive strengths of backfill at different ages using Box–Behnken Design in response- surface methodology. There are many research studies on the mix proportion and performance of backfill materials using response-surface methodology [20,21]. Huang et al. [22] predicted the optimal proportion of mine backfill materials based on a neural-network-genetic algorithm; Feng et al. [23] studied the compressive strength of multi-source CSW by combining orthogonal test and BP neural network; Li et al. [24] optimized the proportion of the tailings-cemented backfill using game-tree-analysis methodology, based on the test results of the backfill strength in the laboratory. Most of the research objects above are backfill materials from non-coal-mine solid waste, while a small number of studies focus on backfill materials from coal mine, and the materials used are only common solid wastes, such as CG and FA. There are a few applications and studies on multi-source CSWs, such as FGD gypsum and GCS as components of backfill materials. In addition, most of the research literature on the mix proportion of backfill materials does not consider backfilling cost, and the optimal mix proportion of the backfilling material obtained is low in strength, which is not suitable for the backfilling field in coal mines. In modern mining and backfilling, backfilling cost has become a factor that cannot be ignored. The preparation cost of backfill materials is an important part of the backfilling cost. Therefore, based on common-solid-waste backfill materials in coal mines, coal-chemical solid wastes, such as GCS, are added as backfilling-material components in this paper, and a mine-backfill-material-proportion scheme that meets the requirements of engineering applications and has relatively optimal comprehensive indicators is proposed, which helps to reduce the blindness of CSW used in underground backfilling projects. The preparation of multi-source CSW into backfill materials and backfilling them into the underground will help large-scale disposal of solid waste in China’s coal power and coal-chemical bases, and can also enable coal mines to obtain backfilling benefits, such as mining coal pillars, reducing surface subsidence, and improving mine ecological benefits. When preparing backfill materials from multi-source CSW, it is necessary to consider the mix-proportioning scheme of backfill materials. In engineering applications, the backfill-material-proportioning scheme should not only consider fluidity and early strength, but also consider material cost. It can be seen that the mix-proportion-optimization scheme of backfill material is essentially a multi-factor and multi-objective optimization problem [11,25,26], and it is suitable to adopt the multi-objective, decision-making model.

In order to obtain the mix proportion of backfill materials with optimal-comprehensive-evaluation indexes that are suitable for engineering application, in this paper, the variation law of slump-flow, bleeding rate, and early strength of the backfill material from CSW, composed of CG, FA, FGD gypsum, and GCS, is studied using single- factor transformation method. The mix-proportion optimization of CSW backfill materials is studied using a multi-objective, decision-making model, and the optimum mix proportion of backfill materials whose fluidity and early strength meet the requirements of mine backfilling and whose cost is the lowest is obtained. Finally, the microstructure of the backfill material with optimum mix proportion is studied.

## 2. Multi-Objective Decision-Making Model

In addition to improving the backfilling amount and effect, backfilling cost should be reduced as much as possible in the engineering practice of mine backfilling. The mix proportion of backfill materials is the key to affect the performance of the mine-backfilling body, and further affects the backfilling cost, which involves fluidity, strength, and material cost. Mix-proportion optimization of backfill materials is a multi-factor and multi-objective optimization problem. Therefore, the application of multi-objective, decision-making theory can comprehensively reflect the engineering-application feasibility for mix proportion of backfill slurry, and more effectively select the mix-proportion scheme suitable for mine application [11,25,26,27,28].

For the multi-objective, decision-making model, the objective eigenvalue matrix *X* is first established, and *m* indicators are used to comprehensively evaluate *n* schemes [26,28,29]. The target eigenvalue matrix *X* is:

$$X=\left[\begin{array}{cccc} x_{11} & x_{12} & \cdots & x_{1n}\\ x_{21} & x_{22} & \cdots & x_{2n}\\ \vdots & \vdots & \vdots & \\ x_{m1} & x_{m2} & \cdots & x_{mn} \end{array}\right]={\left(x_{ij}\right)}_{m\times n}$$

For scheme *j*, vector *x_j_* can be used to represent the eigenvalues of *m* indicators; then,

$$x_{j}={\left[x_{1j},\;x_{2j},\ldots ,\;x_{mj}\right]}^{T}$$

According to the types of indicators, the target eigenvalue matrix is normalized by Equations (1)–(3) to eliminate the influence of different dimensions of the evaluation indicators, and the target-relative-superiority matrix *R* is as follows:

$$R=\left[\begin{array}{cccc} r_{11} & r_{12} & \cdots & r_{1n}\\ r_{21} & r_{22} & \cdots & r_{2n}\\ \vdots & \vdots & \vdots & \\ r_{m1} & r_{m2} & \cdots & r_{mn} \end{array}\right]={\left(r_{ij}\right)}_{m\times n}$$

The larger-the-better type:

(1) $$r_{ij}=\frac{x_{ij}-min\left(x_{ij}\right)}{max\left(x_{ij}\right)-min\left(x_{ij}\right)}$$

The moderate-intermediate type:

(2) $$r_{ij}=1-\left|\frac{x_{ij}-y_{i}}{max\left(x_{ij}\right)-min\left(x_{ij}\right)}\right|$$

The smaller-the better type:

(3) $$r_{ij}=\frac{max\left(x_{ij}\right)-x_{ij}}{max\left(x_{ij}\right)-min\left(x_{ij}\right)}$$

where, *r_ij_* represents the vector in the target-relative-superiority matrix *R*; *x_ij_* represents the target eigenvalue of the *i*-th index of scheme *j*; *y_i_* is the ideal value of the *i-*th index, *i* = 1, 2, …, *m* and *j* = 1, 2, …, *n*.

**Figure 1:**
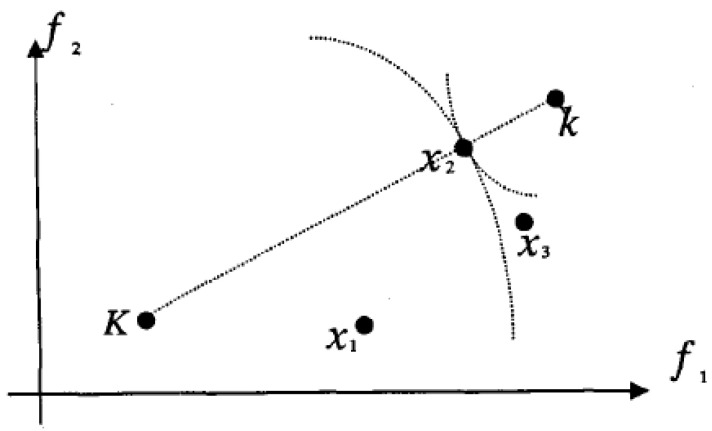
Distance in 2D metric space.

In the paper, the ideal solution and the worst solution are set up as benchmarks to measure the proximity of other feasible solutions to the two, that is, sorting and optimization are performed according to the distance between the optimal solution and the worst solution from the evaluation object, as shown in Figure 1.

The relative superiority of an ideal scheme can be expressed as a vector *k* = (*k*_1_, *k*_2_,..., *k*_n_) = (1, 1,..., 1), and that of the worst scheme can be expressed as a vector *K* = (*K*_1_, *K*_2_,..., *K*_n_) = (0, 0,..., 0). The ideal and worst schemes are used as a basis to measure the proximity of other feasible schemes to the two.

The distance from the scheme *x_i_* (*i* = 1, 2,…, *m*) to the ideal scheme is as follows:

(4) $$l_{ik}=\sum_{i=1}^{n}{\left[w_{i}\left(1-r_{ij}\right)\right]}^{2}$$

The distance from the scheme *x_i_* (*i* = 1, 2,…, *m*) to the worst scheme is as follows:

(5) $$l_{ik}=\sum_{i=1}^{n}{\left[w_{i}(r_{ij}-0)\right]}^{2}$$

where, *w_i_* represents the weight of the *i*-th evaluation index.

The following rules can be selected for the optimization of decision-making schemes:

(1) The closer the decision-making scheme *x*_i_ is to the ideal scheme, the better it will be.

The single-objective-optimization model is established:

$$\{\begin{array}{c} min\;e\left(w\right)=\overset{n}{\underset{i=1}{\sum }}l_{ik}\left(w\right)\\ st:\;w_{j}\geq 0,\;\overset{m}{\underset{j=1}{\sum }}w_{j}=1\; \end{array}\;$$

where, $l_{ik}\left(w\right)=\sum_{j=1}^{m}w_{j}{\left[r_{ij}-1\right]}^{2}$.

To find the solution of the model, the Lagrange function is constructed:

$$L\left(w,\xi \right)=\overset{n}{\underset{i=1}{\sum }}\overset{m}{\underset{j=1}{\sum }}w_{j}{\left(r_{ij}-1\right)}^{2}+\xi \left(\overset{m}{\underset{j=1}{\sum }}w_{j}-1\right)$$

Let $\{\begin{array}{c} \frac{\partial L}{\partial w_{j}}=0\\ \frac{\partial L}{\partial \xi }=0 \end{array}$, the optimal solution is solved to obtain $w=\left(w_{1},\;w_{2},\ldots ,w_{m}\right)$, which is substituted into $l_{ik}$. The scheme *x*_i_ is sorted according to the value of $l_{ik}$, and the scheme corresponding to the minimum value of $l_{ik}$ is the optimal scheme.

(2) The farther away the decision-making scheme *x*_i_ is from the worst scheme, the better it will be.

The single-objective-optimization model is established:

$$\{\begin{array}{c} max\;e\left(w\right)=\overset{n}{\underset{i=1}{\sum }}l_{ik}\left(w\right)\\ st:\;w_{j}\geq 0,\;\overset{m}{\underset{j=1}{\sum }}w_{j}=1\; \end{array}$$

where, $l_{ik}\left(w\right)=\sum_{j=1}^{m}w_{j}{\left(r_{ij}\right)}^{2}$. 

To find the solution of the model, the Lagrange function is constructed:

$$L\left(w,\xi \right)=\overset{n}{\underset{i=1}{\sum }}\overset{m}{\underset{j=1}{\sum }}w_{j}{\left(r_{ij}\right)}^{2}+\xi \left(\overset{m}{\underset{j=1}{\sum }}w_{j}-1\right)$$

The method for solving the model is the same as above. The scheme *x*_i_ is sorted according to the value of $l_{ik}$, and the scheme corresponding to the maximum value of $l_{ik}$ is the optimal scheme.

(3) The decision-making scheme *x*_i_ should be as close to the ideal scheme as possible and far away from the worst scheme.

Since the standards of rules (1) and (2) are different, the same results may not be obtained when using these two rules to sort the decision-making schemes, and even the final results may be contradictory. For this reason, a new rule is established, that is, the decision-making scheme should be as close to the ideal scheme as possible and far away from the worst scheme. This rule is chosen in this paper to select the optimal solution.

The optimal solution of the decision-making scheme must satisfy *x_i_*, and must also have the smallest degree of dispersion with *k* and *K*, that is, the sum of the weighted distance squared of *x_i_* belonging to *K* and that of *x_i_* belonging to *k* reaches a minimum value, as shown in Equation (6). Finally, the relatively optimal scheme is determined by comparing the relative superiority of each scheme [25,26].

(6) $$u_{j}=1/\left\{1+\frac{l_{ik}}{l_{iK}}\right\}$$

Weights are the key to multi-objective, decision-making models. The weight is determined by the importance of the evaluation index, and the weight matrix is established as follows:

$$w=\left[\begin{array}{cccc} w_{1} & w_{2} & \cdots & w_{i} \end{array}\right]$$

where *w_i_* represents the weight of the *i*-th evaluation index, *w_i_* ≥ 0 and $\sum_{i=1}^{m}w_{i}=1$. 

**Figure 2:**
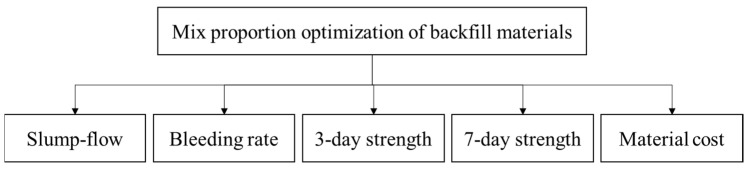
Hierarchy of mix-proportion optimization.

The weight of the evaluation index can be determined using the Analytic Hierarchy Process [25,30,31]. First, the research objects in this paper are divided into two levels to simplify the calculation, as shown in Figure 2

Second, the judgment matrix *A* is established using the general 1–9 scale method:

$$A=\left[\begin{array}{cccc} a_{11} & a_{12} & \ldots & a_{1n}\\ a_{21} & a_{22} & \ldots & a_{2n}\\ \ldots & \ldots & \ldots & \ldots \\ a_{n1} & a_{n2} & \ldots & a_{nn} \end{array}\right]$$

Then, the weight vector is solved by the sum method and matrix normalization, namely:

(7) $$w_{i}=\frac{1}{n}\sum_{j=1}^{n}\frac{a_{ij}}{\sum_{k=1}^{n}a_{kj}}$$

To determine whether matrix *A* meets the consistency requirements, consistency inspection is also required, as follows:

Consistency index:

(8) $$CI=\frac{\lambda_{max}-n}{n-1}$$

where *λ_max_* is the maximum eigenvalue of the judgment matrix *A*, which can be calculated by $AW=\lambda_{max}W$.

Consistency ratio:

(9) $$CR=\frac{CI}{RI}$$

where *RI* can be found in the random-consistency-index table. When *CR* < 0.1, it is considered that judgment matrix *A* meets the consistency requirements.

## 3. Materials and Methods

### 3.1. Experimental Materials

**Figure 3:**
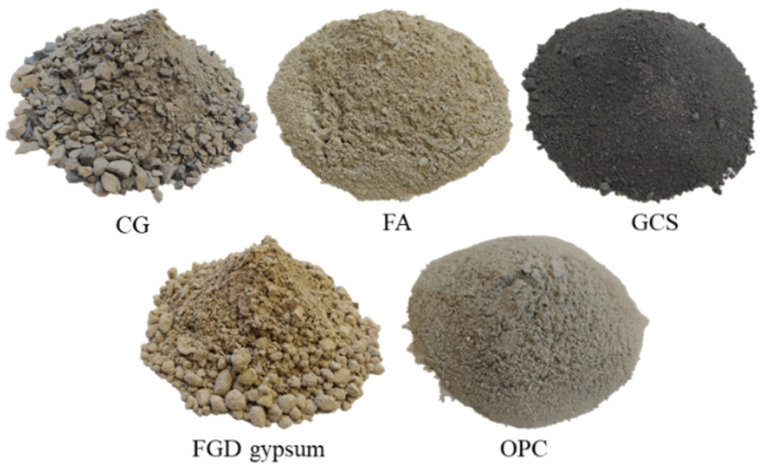
Experimental materials.

FA, GCS, FGD gypsum, and CG are used as experimental materials, and a small amount of OPC is added, as shown in Figure 3.

#### 3.1.1. FA

**Figure 4:**
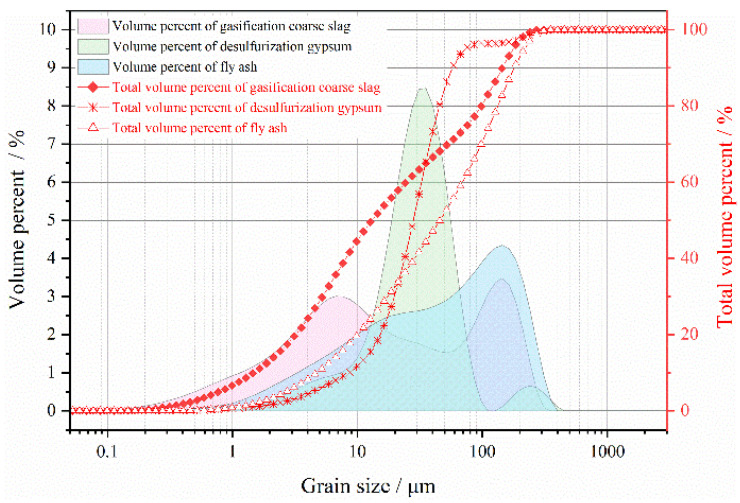
Particle-size distribution of FA, GCS, and FGD gypsum.

The FA in the experiment is secondary ash, and its fineness is 20 (that is, the ratio of FA particles passing through a 45 μm square-hole sieve is 80%). The loss on ignition is 2.5%, the specific surface area is 481.2 m^2^/kg, the moisture content is less than 1%. It is gray-white and powdery. The particle-size range of FA is 0.3–300 μm, and the particle size below 100 μm accounts for about 85% of the total range (as shown in Figure 4). The main mineral components of FA are quartz and mullite.

#### 3.1.2. GCS

GCS is black, granular, and has a water content of about 5%. The particle-size range is 0.2–300 μm, and the volume percentage of GCS below 150 μm is about 90%. The main mineral component of the GCS is quartz, which accounts for more than 95% (as shown in Figure 4).

#### 3.1.3. FGD Gypsum

FGD gypsum is dark-yellow, with a moisture content of about 10%. It is massive and can be broken into powder after drying. The particle-size-distribution range is 0.5–400 μm, and the volume percentage of the particle size smaller than 30 μm is 70% (as shown in Figure 4). The main mineral component of FGD gypsum is dihydrate gypsum, the content of which exceeds 95%.

#### 3.1.4. CG

The lithology of CG in the experiment is mainly sandstone and shale, and the main mineral components are quartz, kaolinite, and a small amount of hematite. It is hard, gray-black, and the original CG needs to be crushed and sieved. The CG with a particle size of less than 10 mm is used as the experimental material, and the CG with a particle size of 5–10 mm accounts for more than 50%.

#### 3.1.5. OPC

The grade of OPC produced in Ningxia is P.O. 42.5, gray-white, powdery, with a specific surface area of 358.1 m^2^/kg and a volume percentage of less than 55 μm over 85%.

### 3.2. Solid Waste Mix Proportion Scheme

In order to study the influence of FA, GCS, FGD gypsum, and CG on the fluidity and early strength of backfill materials, the experimental scheme was designed using a single-factor-transformation method. One of the four solid waste materials is used as a variable factor in turn, and its mass ratio in the solid waste material is set to 10%, 17%, 25%, 33%, and 40% in turn, and the ratio of the other three solid wastes is equally divided. The mass concentration of backfill material is 78%, and the OPC content is 2% of the total amount of solid-waste materials. The experimental design scheme is shown in Table 1.

#### 3.2.1. Slump-Flow and Bleeding Rate Test

In this experiment, slump-flow and bleeding rate are used as the fluidity-evaluation indexes of backfill materials. The tool for measuring the slump-flow in the experiment is a small slump bucket. The upper-opening diameter of the bucket is 5 cm, the lower-opening diameter is 10 cm, and the height is 15 cm. The ratio of the standard slump test result to the small-slump-bucket test result is 2.28, which can accurately and effectively obtain the slump-flow of the backfill material [32,33]. GB/T50080-2016 is referred to as the test methods for slump-flow and bleeding rate.

#### 3.2.2. Strength Test

The size of the test mold for backfill-material molding is 70.7 × 70.7 × 70.7 mm (length × wide × high). The curing conditions of the specimens simulate the underground environment. The curing conditions are the temperature of 20 °C, the relative humidity of 85%, and the curing time of 3-day and 7-day, respectively. After curing for the corresponding time, the upper- and lower-end faces of the specimen are ground to reduce the end-face effect. JGJ/T 70-2009 is referred to as the test method of compressive strength.

## 4. Experimental Result

### 4.1. Slump-Flow

**Figure 5:**
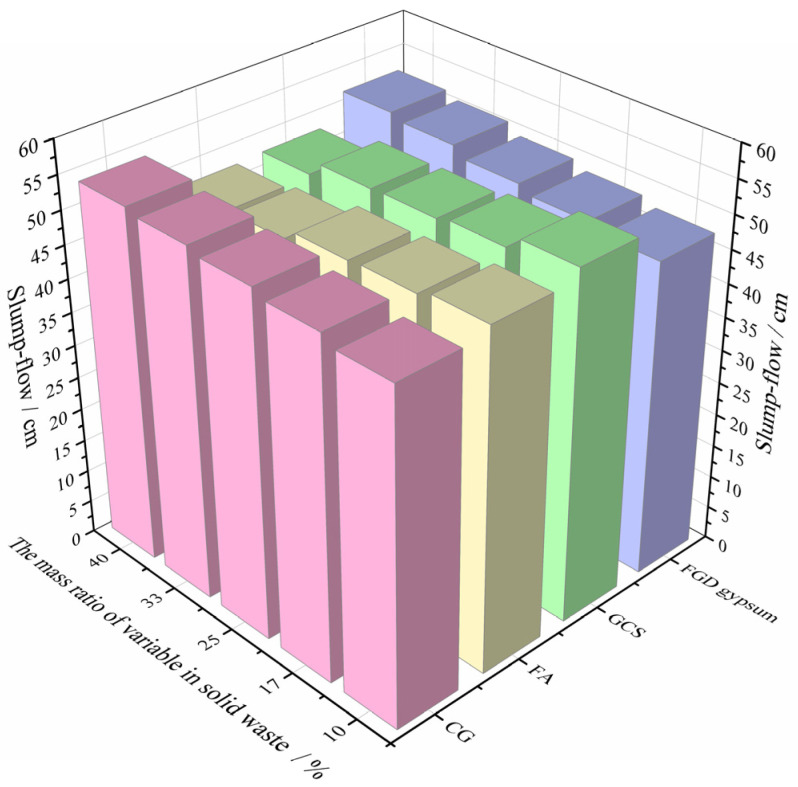
Slump-flow test results.

Through the slump-flow test of backfill materials with different mass ratios of each solid waste, the test results are shown in Figure 5.

It can be seen from Figure 5 that with the increase in CG content, the slump-flow of backfill materials shows a growth trend. When the mass ratio of CG to solid waste increases from 10% to 40%, the slump-flow of backfill material increases from 49.8 cm to 53.5 cm, with an increase of 7.42%. The increase in the content of FGD gypsum also shows a positive effect on the slump-flow of the backfill material. When its mass ratio increases from 10% to 40%, the slump-flow of the backfill material increases from 37.7 cm to 42 cm, which is an increase of 9.01%. The increase of FA and GCS content has a negative impact on the slump-flow of backfill material, that is, when the mass ratio of FA or GCS to solid waste increases from 10% to 40% respectively, the slump-flow of backfill material decreases from 51.2 cm or 52.7 cm to 47.3 cm or 47.7 cm, respectively, with a decrease of 7.62% or 9.49%. It can be seen that as the content of FA and GCS increases, the viscosity of backfill material increases, leading to a reduction of slump-flow.

### 4.2. Bleeding Rate

**Figure 6:**
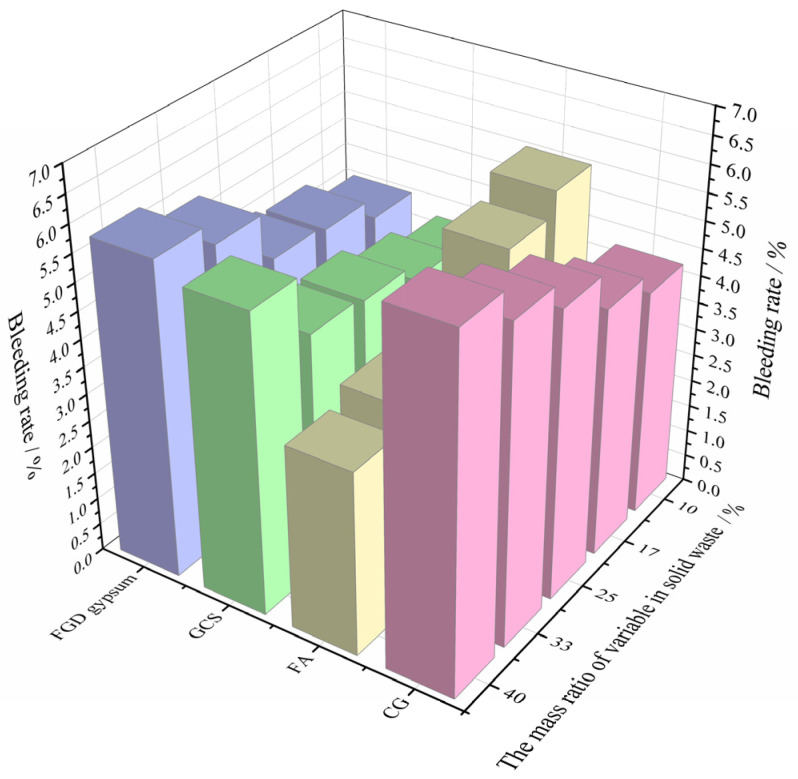
Bleeding-rate test results.

Through the bleeding-rate test of backfill materials with different content of each solid waste, the test results are shown in Figure 6.

According to the relationship between the mass ratio of different solid wastes and the bleeding rate of backfill materials in Figure 6, when the mass ratio of CG, GCS, and FGD gypsum increases from 10% to 40%, respectively, the bleeding rate of backfill materials increases from 4.2%, 3.7%, and 4%, to 6.4%, 5.5%, and 5.7%, respectively, with an increase of 52.38%, 48.65%, and 42.5%, respectively. It can be seen that the increase in the mass ratio of CG, GCS, and FGD gypsum to solid waste has a positive impact on the growth of the bleeding rate of backfill materials. However, the influence of FA on the bleeding rate of the backfill materials shows a negative effect. When the mass ratio of FA increases from 10% to 40%, the bleeding rate of the backfill material decreases from 5.5% to 3.4%, with a decrease of 38.18%.

### 4.3. Early Strength

**Figure 7:**
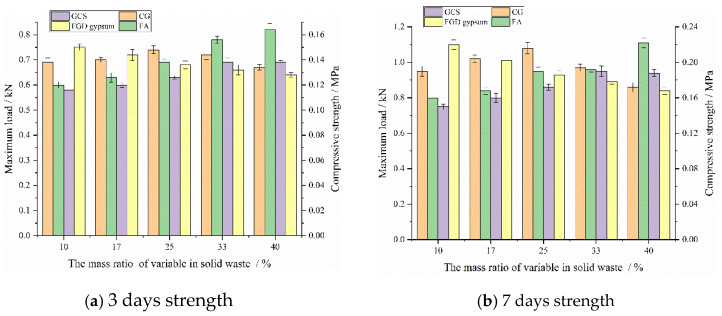
Relationship between different solid-waste proportions and early strength.

The backfill specimens with curing ages of 3 days and 7 days are tested using uniaxial compression, and the relationship between different solid-waste mass ratios and the strength of the backfill specimen is shown in Figure 7.

It can be seen from Figure 7 that when the OPC content is 2%, the early strength of backfill materials with different mass ratios of solid wastes is generally low, and the maximum load in 3-day and 7-day are 0.83 kN and 1.11 kN, respectively, while the corresponding compressive strength is 0.16 MPa and 0.22 MPa. The early strength changes of the backfill materials at 3-day and 7-day are basically the same, but the influence of each solid waste on the early strength of the backfill specimens is different. When the mass ratio of CG is less than 25%, the 3-day and 7-day strengths increases with the increase in the mass ratio of CG. When the mass ratio of CG is more than 25%, the 3-day and 7-day strengths of the backfill material decrease. The maximum loads of 3-day and 7-day with the proportion of CG being 25% are 0.74 kN and 1.08 kN, respectively. The mass ratio of FA increases from 10% to 40%, and the maximum load in 3-day and 7-day increases from 0.6 kN and 0.8 kN, to 0.82 kN and 1.11 kN, respectively, with an increase of 36.67% and 38.75%, demonstrating a significant increase. The mass ratio of GCS increases from 10% to 40%, and the increase of 3-day and 7-day strengths is 22.81% and 26.67%, respectively, indicating that with the increase of the mass ratio of FA and GCS, the 3-day and 7-day strengths of backfill material show a slow increase trend. However, when the mass ratio of GCS is more than 33%, the strengths of 3-day and 7-day do not increase but decline, indicating that the high content of GCS is not conducive to early strength growth. The mass ratio of FGD gypsum increases, and the strengths of backfill materials in 3-day and 7-day decrease slowly. The mass ratio of FGD gypsum increases from 10% to 40%, and the maximum load of backfill materials in 3-day and 7-day decreases from 0.75 kN and 1.1 kN, to 0.64 kN and 0.84 kN, respectively, with a decrease of 14.67% and 23.64%.

### 4.4. Reasonable Proportion of Solid Wastes

According to the literature [11,12,26], the reasonable range of the bleeding rate of backfill material is 5%–20%. It can be seen from Figure 5 that the results are in line with the target eigenvalue in this experiment, as shown in Table 2.

According to Table 2, the target-eigenvalue-matrix *X* is as follows:

$$X_{4\times 8}=\left[\begin{array}{cccccccc} 51.4 & 50.5 & 47 & 51.4 & 52.2 & 52.5 & 53.33 & 54\\ 5.5 & 5.1 & 5.5 & 5.4 & 5.8 & 5.3 & 5.8 & 6.4\\ 0.12 & 0.13 & 0.14 & 0.13 & 0.13 & 0.15 & 0.14 & 0.13\\ 0.16 & 0.17 & 0.19 & 0.18 & 0.17 & 0.22 & 0.19 & 0.17 \end{array}\right]$$

The slump-flow indicates the flow distance of the backfill material, which is regarded as the larger-the-better type; within a reasonable range, the bleeding rate belongs to the smaller-the-better type; the early strength belongs to the larger-the-better type. Accordingly, the target eigenvalues are normalized by Equations (1)–(3) to obtain the target- relative-superiority matrix *R*:

$$R_{4\times 8}=\left[\begin{array}{cccccccc} 0.5667 & 0.4167 & 0 & 0.5667 & 0.7 & 0.75 & 0.8833 & 1\\ 0.6923 & 1 & 0.6923 & 0.7692 & 0.4615 & 0.8462 & 0.4615 & 0\\ 0.0012 & 0.2013 & 0.6014 & 0.4013 & 0.2680 & 0.9348 & 0.8015 & 0.4680\\ 0.0008 & 0.1342 & 0.4676 & 0.3009 & 0.1342 & 0.9344 & 0.5676 & 0.2009 \end{array}\right]$$

Slump-flow and bleeding rate can measure fluidity to a certain extent, but the bleeding rate is more focused on measuring the water-retention performance of slurry. It can be seen from the literature [25,34] that, within the reasonable range of the bleeding rate in line with the paste backfill, the influence degree of the slump-flow on fluidity is higher than that of the bleeding rate, and the early strength should also be considered. Therefore, according to relevant literature and combined with relevant knowledge and experience, the order of importance of the impact on fluidity is slump-flow > bleeding rate > 3- day or 7-day strength, and the judgment matrix is constructed as follows:

$$A=\left[\begin{array}{cccc} 1 & 2 & 4 & 4\\ \frac{1}{2} & 1 & 3 & 3\\ \frac{1}{4} & \frac{1}{3} & 1 & 1\\ \frac{1}{4} & \frac{1}{3} & 1 & 1 \end{array}\right]$$

The weight vector is calculated by Equation (4):

$$w=\left[\begin{array}{cccc} 0.4836 & 0.2974 & 0.1095 & 0.1095 \end{array}\right]$$

That is, the weight of each index is slump-flow *w*_1_ = 0.4836, bleeding rate *w*_2_ = 0.2974, 3-day strength *w*_3_ = 0.1095, and 7-day strength *w*_4_ = 0.1095.

The consistency ratio of the judgment matrix A calculated by Equations (5) and (6) is 0.0077 < 0.1, which shows that the judgment matrix A meets the consistency requirements, and the judgment matrix is reasonable.

The relative superiority of the alternative results is calculated by Equation (7), and the results are as follows:

$$u_{j}=\left[\begin{array}{cccccccc} 0.6065 & 0.5742 & 0.1662 & 0.6894 & 0.6841 & 0.9277 & 0.8710 & 0.7043 \end{array}\right]$$

According to the calculation results, the relative superiority of Experiment No. A6 is 0.9277, which is the maximum, so it can be regarded as the scheme with the best comprehensive index. The mix proportion of solid-waste materials in Experiment No. A6 is FA: GCS: FGD gypsum: CG = 25%: 25%: 25%: 25%: 25%. The backfill material with this mix proportion has the best fluidity, that is, the slump-flow is 52.5 cm, the bleeding rate is 5.3%, and the strengths of 3-day and 7-day are 0.15 MPa and 0.22 MPa, respectively.

## 5. Mix Proportion of Backfill Material

The optimal mix proportion of FA, GCS, FGD gypsum, and CG is obtained using a single-factor transformation experiment. However, the strengths of 3-day and 7-day are generally low due to the small amount of OPC added, which cannot meet the requirements of the mine for the strength of the backfill body. Therefore, it is necessary to appropriately increase the proportion of cementitious materials, optimize the proportion of backfill materials, according to the requirements of mine backfilling on fluidity, strength, and cost, and prepare backfill materials with the reasonable proportion that both fluidity and strength meet the requirements of mine backfilling, while the cost is relatively low.

### Experimental Scheme

The optimal mix proportion of FA, GCS, FGD gypsum, and CG is fixed, that is FA:GCS:FGD gypsum:CG = 25%:25%:25%:25%. The fluidity and early strength of the backfill materials increase by changing the mass concentration and cement content. The design-mass concentration is 78%, 76%, and 74%, respectively, and the mass ratio of OPC to the total solid waste is 10%, 15%, and 20%, respectively. The specific experimental scheme is shown in Table 3.

## 6. Experimental Result

### 6.1. Slump-Flow and Bleeding Rate

**Figure 8:**
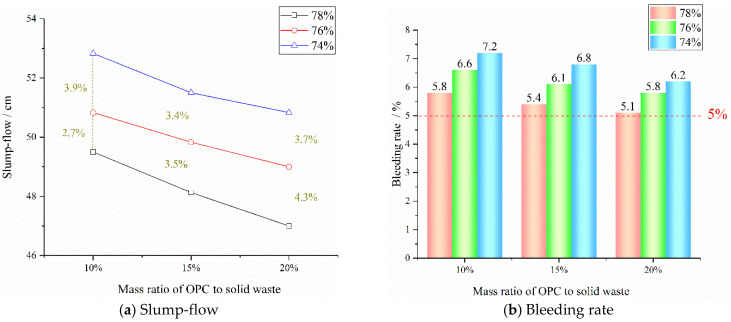
Test results of slump-flow and bleeding rate of backfill materials with different mass concentrations and OPC-mass ratios.

The test results of slump-flow and bleeding rate are shown in Figure 8 to evaluate the influence of mass concentration and OPC content on the fluidity of backfill materials.

It can be seen from Figure 8a that when the mass concentration is the same, with the increase of the mass ratio of OPC to solid waste, the slump-flow of the backfill material gradually decreases. When the mass concentration is 78%, 76%, and 74%, respectively, the slump-flow of backfill materials with 20% of OPC is 2.5 cm, 1.83 cm, and 2 cm lower than that of backfill materials with 10% of OPC, with a decrease of 5.1%, 3.6%, and 3.8%, respectively. When the content of OPC is the same, with the decrease of the mass concentration, the slump-flow of backfill materials increases continuously. When the mass concentration decreases from 78% to 76%, the slump-flow of backfill materials with 10%, 15%, and 20% of OPC increases by 2.7%, 3.5%, and 4.3%, respectively. When the mass concentration decreases from 76% to 74%, the slump-flow of backfill materials with corresponding OPC mass ratios increases by 3.9%, 3.4%, and 3.7%, respectively. When the mass concentration decreases from 78% to 74%, the slump-flow of backfill materials with different OPC mass ratios increases by more than 6.5%. 

It can be seen from Figure 8b that when the OPC content is the same, the bleeding rate of the backfill material gradually increases with the decrease in the mass concentration. When the mass concentration decreases from 78% to 74%, the bleeding rate of backfill materials with OPC mass ratios of 10%, 15%, and 20% increases from 5.8%, 5.4%, and 5.1%, to 7.2%, 6.8%, and 6.2%, respectively, with an increase of more than 20%. It can be seen that the mass concentration has a significant impact on the bleeding rate of backfill materials. When the mass concentration is the same, with the increase of the OPC-mass ratio, the bleeding rate of the backfill material shows a slow downward trend. When the mass proportion of OPC increases from 10% to 20%, the bleeding rate of backfill materials with mass concentrations of 78%, 76%, and 74% decreases from 5.8%, 6.6%, and 7.2%, to 5.1%, 5.8%, and 6.2%, respectively, with a general decrease of 12–14%. It can be seen that the content of OPC has an obvious impact on the bleeding rate of backfill materials. However, when the mass concentration is 78%−74% and the mass ratio of OPC is between 10%−20%, the bleeding rate of backfill materials is more than 5%, which meets the requirement that the bleeding rate of backfill materials in the mine is 5–20%.

To sum up, the influence of mass concentration and OPC content on the slump-flow and bleeding rate of backfill materials shows a negative effect. In the experiment, the bleeding rate of nine groups of backfill materials is more than the minimum limit of 5%, and their slump-flow is more than 47 cm, which meets the requirements of the mine for the fluidity of backfill materials.

### 6.2. Early Strength

**Figure 9:**
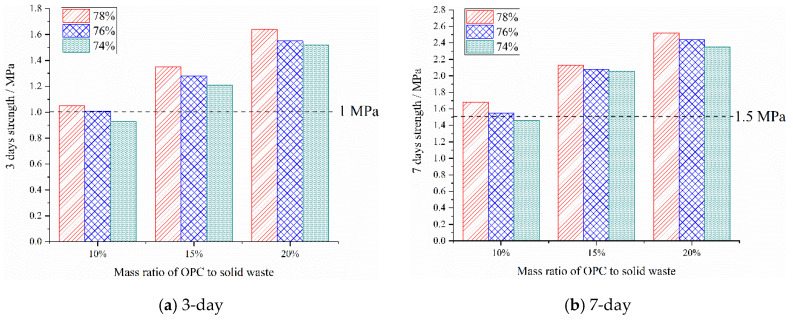
Early-strength test results of backfill materials.

The early-strength results of the backfill specimens cured for 3 days and 7 days are obtained through a compressive-strength test, as shown in Figure 9.

It can be seen from Figure 9a,b that OPC has a significant and positive effect on the early strength of the backfill specimens. As the mass ratio of OPC increases, the early strength of the backfill specimens increases significantly. When the mass ratio of OPC increases from 10% to 20%, the 3-day and 7-day strengths of the backfill specimen with 78% mass concentration increase from 1.05 MPa and 1.68 MPa, to 1.64 MPa and 2.52 MPa, respectively, with an increase of 56.19% and 50%, respectively. It can be seen that the strength of the backfill specimen mainly depends on the OPC content. At the same curing age, different mass concentrations also have a positive effect on the early strength of the backfill specimens, that is, with the increase of mass concentration, the compressive strength of the backfill specimens with the same mass ratio of OPC shows a slow growth trend at 3-day and 7-day. When the mass concentration is 78%, 76%, and 74%, respectively, the 3-day strength of the backfill specimen with a mass ratio of 15% OPC is 1.35 MPa, 1.28 MPa, and 1.21 MPa, and the 7-day strength is 2.13 MPa, 2.08 MPa, and 2.06 MPa, respectively. When the mass concentration decreases from 78% to 74%, the 3-day and 7-day strengths of the backfill specimens decrease by 0.14 MPa and 0.07 MPa, respectively, with a decrease rate of 10.37% and 3.29%, which is a small decrease. It can be seen that the mass concentration has little effect on the early strength of the backfill specimen.

### 6.3. Mix Proportion Optimization

The early strength of backfill body should meet the requirements of rock-stratum control. Literature [19,26] shows that mines require a backfill strength of not less than 1 MPa for three days and not less than 1.5 MPa for seven days. It can be seen from Figure 6 that the 3-day and 7-day strengths of the backfill specimen with a mass concentration of 74% are 0.94 MPa and 1.46 MPa, respectively, which are less than the theoretically required strengths of 1 MPa and 1.5 MPa for 3-day and 7-day, and do not meet the requirements of mine backfilling. Therefore, there are eight experimental groups that meet the 3-day and 7-day strength requirements and fluidity requirements for mine backfill, which are used as alternative results. The slump-flow, bleeding rate, 3-day strength, 7-day strength, and material cost are selected as the evaluation indicators, and the alternative results are optimized. The index values of the alternative results are shown in Table 4. The 3-day strength and 7-day strength belong to the larger-the-better type; the bleeding rate in the range of 5%−20% can be regarded as the smaller-the-better type, the slump-flow belongs to the larger-the-better type; the material cost belongs to the smaller-the-better type, and is calculated by the price of each material required for the unit mass of backfill material. The material cost is mainly the cost of OPC and water, and the solid waste is from the local, ignoring the transportation cost.

The target-eigenvalue matrix *X* obtained according to Table 4 is as follows:

$$X_{5\times 8}=\left[\begin{array}{cccccccc} 49.5 & 48.13 & 47 & 50.83 & 49.83 & 49 & 51.5 & 50.83\\ 5.8 & 5.4 & 5.1 & 6.6 & 6.1 & 5.8 & 6.8 & 6.2\\ 1.05 & 1.35 & 1.64 & 1.01 & 1.28 & 1.55 & 1.21 & 1.52\\ 1.68 & 2.13 & 2.52 & 1.55 & 2.08 & 2.44 & 2.06 & 2.35\\ 43 & 64 & 85 & 45 & 66 & 87 & 68 & 89 \end{array}\right]$$

According to Equations (1)–(3), the target eigenvalues are normalized to obtain the target-relative-superiority matrix:

$$R_{5\times 8}=\left[\begin{array}{cccccccc} 0.5556 & 0.2511 & 0 & 0.8511 & 0.6289 & 0.4444 & 1 & 0.8511\\ 0.5882 & 0.8235 & 1 & 0.1177 & 0.4118 & 0.5882 & 0 & 0.3529\\ 0.0635 & 0.5397 & 1 & 0 & 0.4286 & 0.8571 & 0.3175 & 0.8095\\ 0.1340 & 0.5979 & 1 & 0 & 0.4286 & 0.8571 & 0.5258 & 0.8247\\ 1 & 0.5435 & 0.0870 & 0.9565 & 0.5 & 0.0435 & 0.4565 & 0 \end{array}\right]$$

In this section, the eigenvector method is used to determine the weight vector. In backfilling practice, mining enterprises pay the most attention to backfilling cost, followed by early strength, and liquidity. Therefore, the judgment matrix *A* is established as follows:

$$A=\left[\begin{array}{ccccc} 1 & 3 & \frac{1}{3} & \frac{1}{3} & \frac{1}{5}\\ \frac{1}{3} & 1 & \frac{1}{3} & \frac{1}{3} & \frac{1}{5}\\ 3 & 3 & 1 & 3 & \frac{1}{4}\\ 3 & 3 & \frac{1}{3} & 1 & \frac{1}{4}\\ 5 & 5 & 4 & 4 & 1 \end{array}\right]$$

The weight vector is calculated by Equation (4):

$$w=\left[\begin{array}{ccccc} 0.0964 & 0.055 & 0.2184 & 0.1497 & 0.4805 \end{array}\right]$$

That is, the weight of each index is slump-flow *w*_1_ = 0.0964, bleeding rate *w*_2_ = 0.055, 3-day strength *w*_3_ = 0.2184, 7-day strength *w*_4_ = 0.1497, and backfilling cost *w*_5_ = 0.4805.

The consistency ratio of the judgment matrix *A* calculated by Equations (5) and (6) is 0.0979 < 0.1, which shows that judgment matrix *A* meets the consistency requirements.

The relative superiority of the alternative results is calculated by Equation (7), and the results are as follows:

$$u_{j}=\left[\begin{array}{cccccccc} 0.8067 & 0.5881 & 0.2490 & 0.7635 & 0.5022 & 0.1931 & 0.4235 & 0.1729 \end{array}\right]$$

It can be seen from the above calculation results that the relative superiority of Experiment No. 1 is the largest, 0.8067, which can be regarded as the result with the best comprehensive index. The optimal mix proportion of backfill material is FA: GCS: FGD gypsum: CG = 25%:25%:25%:25%, the mass concentration is 78%, and OPC accounts for 10% of the total solid waste. In order to verify the reliability of the evaluation results, the fluidity and early strength of the backfill materials are tested with the optimal material ratio. The results show that the slump-flow is 50.33 cm, the bleeding rate is 5.5%, and the 3-day and 7-day strengths are 1.03 MPa and 1.65 MPa, respectively. The fluidity and early strength of the backfill materials meet the requirements of the mine.

## 7. Performance of Backfill with Optimal Mix Proportion

### 7.1. Pore Structure

**Figure 10:**
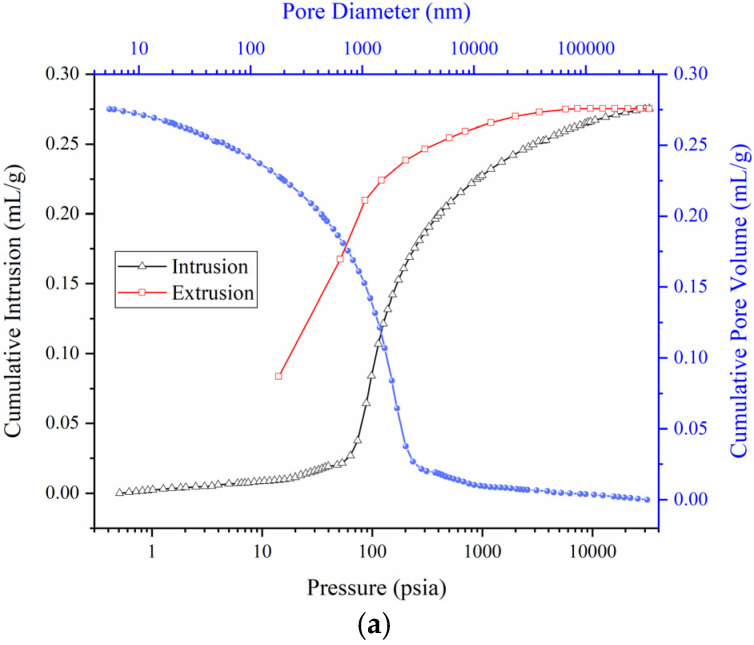

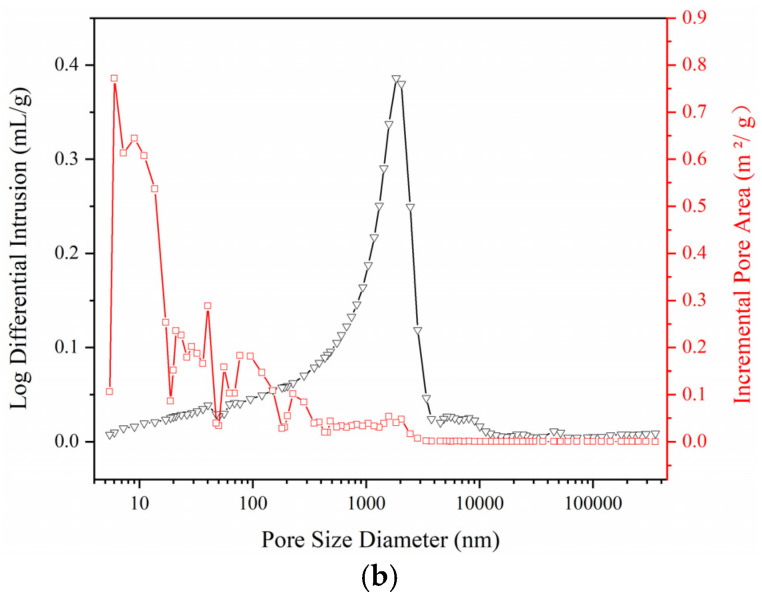
MIP Test Results. (**a**) Mercury injection-removal curve and cumulative-pore-volume curve. (**b**) Log-differential-intrusion curve and incremental-pore-area curve.

The pore structure of the backfill specimen with the optimal mix proportion in Section 6.3 is tested by MIP, and the results are shown in Figure 10.

It can be seen from Figure 10a that when the mercury-injection pressure is less than 60 psia, the mercury-injection amount increases slowly; when the mercury-injection pressure is between 60–400 psia, the mercury-injection amount increases sharply, indicating that the pore-volume content corresponding to the 60–400 psia pressure increases significantly; when the mercury-injection pressure exceeds 400 psia, the increase in the amount of mercury injection slows down, and the mercury enters into smaller pores. During the mercury-removal process, the mercury-removal curve deviates significantly from the mercury-injection curve, and is always above the mercury-injection curve, indicating that there is mercury retention in the mercury-removal process, which is due to the existence of dense narrow-neck closed pores inside the specimen. From the cumulative-pore-volume curve, it can be seen that the pore diameter in the specimen is in the range of 5–4 × 10^5^ nm. When the pore diameter is less than 3000 nm, the cumulative pore volume decreases sharply in a parabolic manner. When the pore diameter is greater than 3000 nm, the cumulative pore volume decreases gradually, indicating that the pore volume is mainly distributed in the pores with a pore diameter of less than 3000 nm. In Figure 10b, the pore-size-distribution curve shows a single-peak shape in the pore-diameter range of 400–3000 nm, and the pore volume changes most significantly. It can be seen that there is a large distribution of pore diameters in the range of 400–3000 nm, and the pore volume with a pore diameter of about 2000 nm is the largest. According to the pore-size-division method of Hodot [35], there are a few micropores and transition pores in the backfill specimen, mainly medium and large pores. The pore-surface-area increment is generally the largest when the pore diameter is less than 20 nm, and the peak value appears at the pore diameter of 6 nm, indicating that micropores have the largest contribution to the pore- surface area.

### 7.2. Micromorphology and Composition

**Figure 11:**
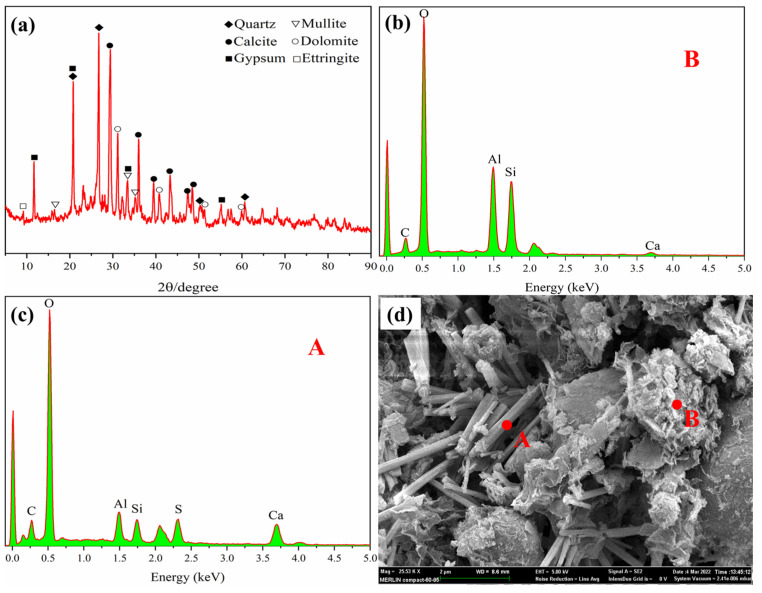
SEM and XRD test results. (**a**) XRD test result. (**b**) The EDS spectrum of point B in the SEM diagram. (**c**) The EDS spectrum of point A in the SEM diagram. (**d**) SEM test result.

Micromorphology and mineral composition of the backfill specimen with the optimal mix proportion are tested by SEM-EDS and XRD, and the results are shown in Figure 11.

The XRD diagram shows that the main mineral phases in the backfill specimen are calcite (CaCO_3_), mullite (2Al_2_O_3_·SiO_2_), gypsum (CaSO₄·2H_2_O), ettringite (3CaO·Al_2_O_3_·3CaSO_4_·32H_2_O), quartz (SiO_2_), and dolomite (CaMg(CO_3_)_2_). The number and intensity of characteristic peaks for quartz, dihydrate gypsum, and calcite are large, indicating that their content is relatively high and their crystallinity is good. It can be seen from the SEM images that there are many CSW particles that do not participate in the hydration reaction, such as spherical-FA particles. In addition, there are some clastic particles in the backfill specimen. According to EDS-point scanning, the clastic particles mainly contain O, Al, and Si. It is judged by XRD that they contain a lot of mullite and a small amount of calcite. Some short rod-shaped substances are interspersed in the voids of solid-waste particles, and the main elements contained therein are O, Al, S, Ca, Si, C, etc. The mole-fraction ratio of Ca and S is about 2. It is judged that the short rod-shaped substances are ettringite, which is the hydration product of OPC, indicating that the hydration product of CSW is mainly OPC, and there is no large amount of chemical reaction between CSW. The reason why C and Si elements are detected in ettringite is that part of Al_2_O_3_ is replaced by SiO_2_ and part of CaSO_4_ is replaced by CaCO_3_.

## 8. Discussion

In this paper, CG, FA, FGD gypsum, and GCS are used as backfill-material components; slump-flow, bleeding rate, material cost, 3-day strength, and 7-day strength are taken as evaluation indicators to optimize the backfill-material mix proportion using a multi-objective, decision-making method, and a group of backfill-material-mix-proportion schemes with optimal comprehensive indexes are obtained. This study helps to reduce the blindness of CSW used in underground-backfilling projects and achieve precise backfilling of solid waste. In addition, the backfill-material-mix-proportion scheme provides a basic theory and scientific guidance for the implementation of backfill engineering. It is expected that the implementation of the backfill-material-proportion results will help with large-scale disposal of CSW in coal-power and coal-chemical bases, and obtain backfilling benefits, such as mining coal pillar and reducing surface subsidence.

However, this study still has some limitations. First, when AHP is used to construct the judgment matrix, the importance between two indicators is determined by references and combining the knowledge and experience of the judges, so it has a certain degree of subjectivity, which is the deficiency of AHP. Second, although the backfill-material-proportion scheme with the best comprehensive index is obtained, the proportion of cement to solid waste in this scheme has reached 10%. For some mines with poor production efficiency, the high proportion of cement leads to an increase in backfilling costs, which may lead to coal mines being reluctant to adopt this backfill-material ratio and backfilling method. Therefore, the application range of the backfill-material-proportion scheme obtained in this study has certain limitations, and it is more suitable for mining coal pillars under buildings, railways, and water bodies to prolong the service life of mines. Third, the backfill-material mix proportion obtained in this study is only from the laboratory, and has not been applied in engineering. The reliability of the backfill- material ratio has only been verified in the laboratory, but not in engineering practice. This is a shortcoming of this study and a practical problem faced by many scientific studies.

This study not only provides an idea for research on the proportion optimization of backfill materials, but also provides a backfilling scheme for large-scale disposal of multi-source CSW discharged from coal-power and coal-chemical bases. This paper is only a piece of preliminary research on the use of CSW for underground backfilling, and there is still a lot of work to be done; future research directions include environmental risk assessment of CSW as a backfill material, the enhancement mechanism of CSW as a backfill material, microstructure evolution and multi-field coupling mechanism of CSW-backfill bodies under the action of underground strata. 

## 9. Conclusions

The slump-flow of backfill material is positively correlated with the content of CG and FGD gypsum, and negatively correlated with the content of FA and GCS. When the content of CG, GCS, and FGD gypsum increases, the bleeding rate of the backfill material decreases, while the increase in the FA content shows a negative effect on the bleeding rate. The increase in FA and GCS content has a positive impact on the early strength of backfill materials, while FGD gypsum has the opposite effect; 25% of CG content is the turning point from positive effect to negative effect on the early strength of backfill materials.

The slump-flow, bleeding rate, and strengths of three days and seven days are taken as evaluation indexes; the optimal mix proportion of solid-waste materials is FA:GCS:FGD gypsum:CG = 25%:25%:25%:25%. Furthermore, by changing the OPC content and mass concentration, it is concluded that OPC accounts for 10% of the total solid waste and the mass concentration is 78%, which is in line with the requirements of mine backfilling and has the best evaluation index.

There are dense, narrow-necked, closed pores in the backfill specimens, and the pore diameter range is 5–4 × 10^5^ nm. There are a few micropores and transition pores, mainly medium and large pores. The hydration reaction of OPC is the main component in the backfill specimens, and there is no large amount of chemical reaction between CSWs. In the hydration-product-ettringite in CSW, part of Al_2_O_3_ is replaced by SiO_2_ and part of CaSO_4_ is replaced by CaCO_3_.

## Figures and Tables

**Table 1:** Solid-waste, mass-ratio experiment scheme.

Variable Factor	Mass Concentration	FA:GCS:FGD Gypsum:G	Mass Ratio of OPC to Solid Waste
FA	78%	10%:30%:30%:30%	2%
		17%:27.7%:27.7%:27.7%	
		25%:25%:25%:25%	
		33%:22.3%:22.3%:22.3%	
		40%:20%:20%:20%	
GCS	78%	30%:10%:30%:30%	2%
		27.7%:17%:27.7%:27.7%	
		25%:25%:25%:25%	
		22.3%:33%:22.3%:22.3%	
		20%:40%:20%:20%	
FGD gypsum	78%	30%:30%:10%:30%	2%
		27.7%:27.7%:17%:27.7%	
		25%:25%:25%:25%	
		22.3%:22.3%:33%:22.3%	
		20%:20%:40%:20%	
CG	78%	30%:30%:30%:10%	2%
		27.7%:27.7%:27.7%:17%	
		25%:25%:25%:25%	
		22.3%:22.3%:22.3%:33%	
		20%:20%:20%:40%	

**Table 2:** Target-characteristic values of solid-waste-proportioning scheme.

No.	FA:GCS:FGD Gypsum:CG	Slump-Flow/cm	Bleeding Rate/%	3-Day Strength/MPa	7-Day Strength/MPa
A1	10%:30%:30%:30%	51.4	5.5	0.12	0.16
A2	17%:27.7%:27.7%:27.7%	50.5	5.1	0.13	0.17
A3	20%:40%:20%:20%	47	5.5	0.14	0.19
A4	22.3%:22.3%:33%:22.3%	51.4	5.4	0.13	0.18
A5	20%:20%:40%:20%	52.2	5.8	0.13	0.17
A6	25%:25%:25%:25%	52.5	5.3	0.15	0.22
A7	22.3%:22.3%:22.3%:33%	53.33	5.8	0.14	0.19
A8	20%:20%:20%:40%	54	6.4	0.13	0.17

**Table 3:** Experimental scheme of different mass concentrations and OPC contents.

No.	Mass Concentration	FA:GCS:FGD Gypsum:CG	Mass Ratio of OPC to Solid Waste
1	78%	25%:25%:25%:25%	10%
2	78%	25%:25%:25%:25%	15%
3	78%	25%:25%:25%:25%	20%
4	76%	25%:25%:25%:25%	10%
5	76%	25%:25%:25%:25%	15%
6	76%	25%:25%:25%:25%	20%
7	74%	25%:25%:25%:25%	10%
8	74%	25%:25%:25%:25%	15%
9	74%	25%:25%:25%:25%	20%

**Table 4:** Target eigenvalues of the alternative scheme.

Index	Slump-Flow/cm	Bleeding Rate/%	3-Day Strength/MPa	7-Day Strength/MPa	Cost/CNY
Weight	0.1	0.05	0.2	0.15	0.5
1	49.5	5.8	1.05	1.68	43
2	48.13	5.4	1.35	2.13	64
3	47	5.1	1.64	2.52	85
4	50.83	6.6	1.01	1.55	45
5	49.83	6.1	1.28	2.08	66
6	49	5.8	1.55	2.44	87
7	51.5	6.8	1.21	2.06	68
8	50.83	6.2	1.52	2.35	89

## Data Availability

The data used in this research have been properly cited and reported in the main text.
